# Computational Oncology in the Multi-Omics Era: State of the Art

**DOI:** 10.3389/fonc.2020.00423

**Published:** 2020-04-07

**Authors:** Guillermo de Anda-Jáuregui, Enrique Hernández-Lemus

**Affiliations:** ^1^Computational Genomics Division, National Institute of Genomic Medicine, Mexico City, Mexico; ^2^Cátedras Conacyt Para Jóvenes Investigadores, National Council on Science and Technology, Mexico City, Mexico; ^3^Center for Complexity Sciences, Universidad Nacional Autónoma de México, Mexico City, Mexico

**Keywords:** multi-omics analysis, computational oncology, data integration, cancer complexity, machine learning, network science

## Abstract

Cancer is the quintessential complex disease. As technologies evolve faster each day, we are able to quantify the different layers of biological elements that contribute to the emergence and development of malignancies. In this multi-omics context, the use of integrative approaches is mandatory in order to gain further insights on oncological phenomena, and to move forward toward the precision medicine paradigm. In this review, we will focus on computational oncology as an integrative discipline that incorporates knowledge from the mathematical, physical, and computational fields to further the biomedical understanding of cancer. We will discuss the current roles of computation in oncology in the context of multi-omic technologies, which include: data acquisition and processing; data management in the clinical and research settings; classification, diagnosis, and prognosis; and the development of models in the research setting, including their use for therapeutic target identification. We will discuss the machine learning and network approaches as two of the most promising emerging paradigms, in computational oncology. These approaches provide a foundation on how to integrate different layers of biological description into coherent frameworks that allow advances both in the basic and clinical settings.

## 1. Cancer: The Complex Disease

**Figure 1:**
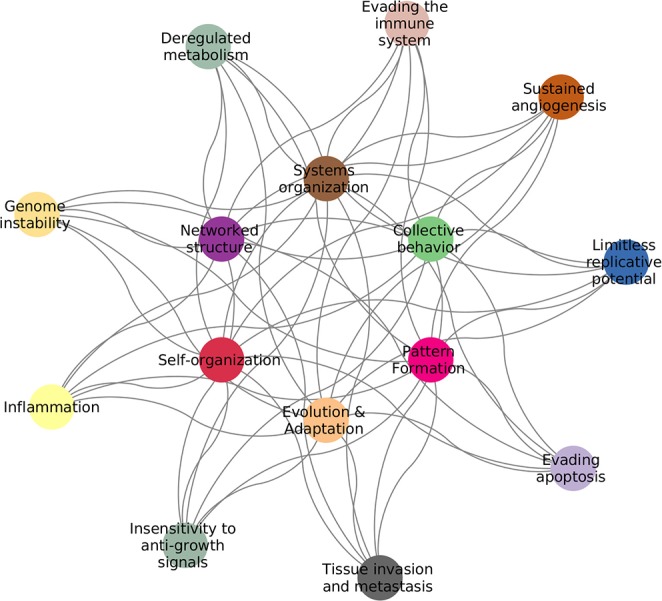
Hallmarks of cancer complexity. The defining features of cancer (3, 4) are intrinsically connected to the defining features of complex systems (2).

Cancer is by now widely accepted to be the quintessential complex disease: a proper description of the pathological phenotype can only be achieved by properly integrating the myriad of interconnected biological elements and their relationships with their environment (1). As a complex system, cancer exhibits features, such as: emergent patterns, adaptive and collective behaviors, self-organization, non-linear dynamics, and interactions forming complex networks (2). Examples of these can be found in the *Hallmarks of Cancer* (3, 4), as seen in Figure 1.

**Figure 2:**
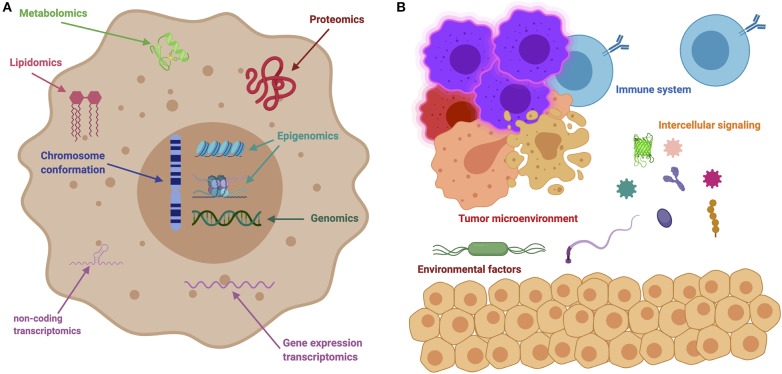
The many levels of interactions found in a cancer system. **(A)** Depicts intracellular interactions that can be measured via the different omic technologies, such as genomics, transcriptomics, metabolomics, lipidomics, and so on. **(B)** Shows intercellular interactions, such as the ones orchestrated through immune responses, microbial interactions (metagenomics) and other instances of cell-cell interactions.

On a system-wide fashion, every tumor is involved in interactions with non-cancer elements: such as gene-environment interactions (GxE) (5), micro-environmental interactions (6), and those with the immune system (7); intercellular interactions within the tumor environment (8); and intracellular interactions, such as transcriptional regulation and gene co-expression (9, 10), signaling (11, 12) and metabolic pathways (13, 14), as well as protein interactions (15). These are exemplified in Figure 2. It soon becomes evident that a major source of cancer complexity lies on the many layers of interacting elements involved in the phenomenon.

## 2. The Multi-Omics Paradigm

### 2.1. Multi-Omics in a Nutshell

Multiomics is the name given to the modelization approach in biology hat makes use of more than one of the current high-throughput biomlecular experimental techniques (a.k.a. omics) in order to characterize biological systems at the phenomenological level. It is understood that every omic contributes on a specific fashion to shape the actual biological phenotype under study. For this reason, it has become evident that there is a need for integrating frameworks to gather and organize the knowledge gained with each experimental approach into mechanistic or semi-mechanistic descriptions of the biological phenomenon. This issue has been deemed particularly relevant for the study of complex phenotypes, such as cancer tumors (16).

**Figure 3:**
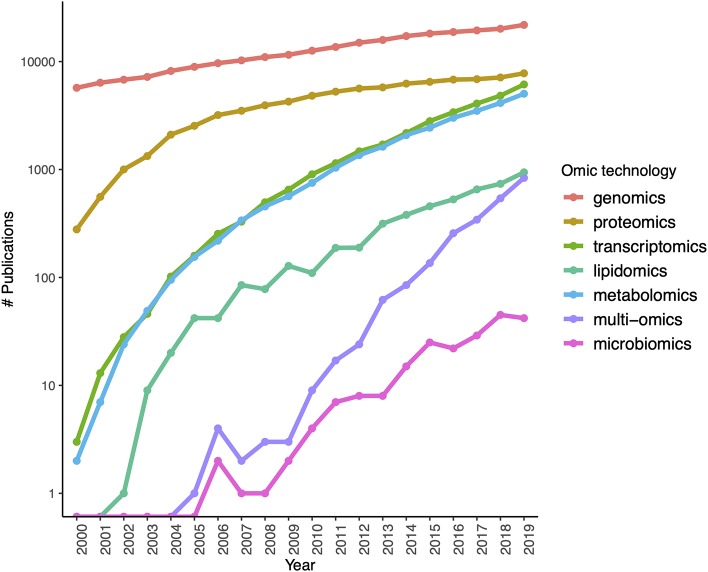
Growth of interest in omics technologies in the twenty-first century: the number of Pubmed publications mentioning each omic technology in its title or abstract measured yearly since the year 2000.

The rapid development of sequencing strategies as well as genotyping and expression microarrays led to the development of gene models to account for the molecular aspects of biology at the whole cellular level (and even at the organ and organism scales). The coming of age and popularization (driven by an almost exponential lowering of the costs) of next gen sequencing techniques leads to an explosion of new approaches to understand complex phenotypes that in turn have sped up the rise of high throughput proteomics, metabolomics catching up. Single cell technologies and a number of arising sequence based approaches (ChIP-seq, ATAC-seq) are becoming usual tools of biomedical and in particular cancer research (see Figure 3, for an account of the fastly increasing number of PubMed publications based on these omic tools).

In spite of this, the integrative approach to multi-omic modeling is far from trivial due to the broad diversity of data types, dynamic ranges and sources of experimental and analytical errors characteristic of each omic. In spite of these facts, a number of approaches to multi-omic integration have been proposed [see, for instance, discussions in Hernández-Lemus (17, 18)]. Said approaches make use of tools from statistics, probability, machine learning and network science to classify, explore and provide guidelines for feature selection and their application is very much rooted in the tenets of systems biology.

The systematic study of cancer given by multi-omics is founded on the acknowledgment of a contribution of many different factors in the development and maintenance of the malignant state, including genetic aberrations, epigenetic alterations, changes in the response to cellular signaling, metabolic alterations, and beyond (19). Hence, by analyzing cancer as a complex pathology, the systems biology paradigm tries to gain insight into the molecular origins of the disease by looking at the diverse contributions, from DNA mutations (both germline and somatic), to deregulation of the gene expression programmes, the phenomenon of hormone disruption, that may or not be supplemented by metabolic abnormalities, and aberrant pathway signaling.

Cancer is also a multiscale pathology, aside from the biomolecular events just mentioned there is the influence of the environment and lifestyle that is known to be able to modify the onset, development, and outcome of tumors and their metastases. Multiomic analysis under a systems biology framework makes possible to use the unprecedented power of current high-throughput molecular and computational tools to draw a more complete figure of the different players in tumorigenesis and tumor establishment. At the same time, it may provide us with new instruments and strategies useful in basic and clinical research laboratories, but also in translational medicine and therapeutic endeavors.

These different levels of description have been independently studied for years. However, even if the advent of high-throughput technologies has permitted the development of systems biology, system-level models (conforming the theoretical foundations of these multiomic studies) are still under development.

### 2.2. The Systems Biology Framework

In essence, the foundational basis of systems biology is that of considering biological phenomena as systems, i.e., constructs formed by a large number of complex molecular and environmental components interacting at different levels to shape the functional features of said system. Tumor behavior, for instance, is determined by a combination of changes in genomic information that may (or may not) be associated with abnormal gene expression profiles; affecting protein abundance, but also modifying protein structure and folding, as well as supramolecular assembly. Changes in the regulatory patterns may also affect cell signaling mechanisms; and their responses. Hence, the complex interaction of nucleic acids and proteins in replication, transcription, metabolic, and signaling networks are considered the ultimate causes for the functioning (or misfunctioning, if preferred) of the tumor cell. We can notice that these are interdependent phenomena that cannot be treated separately, hence the need for integrative methodologies.

Another pivotal challenge in contemporary studies undertaken following a systems biology view is hence data integration. Data integration allows for the understanding of the enormous datasets generated by experimental multi-omics. This is indeed a highly non-trivial task, since just the data management of such large amounts of information represents a challenge that has been called the big data paradigm.

## 3. The Roles of Computation in the Age of Cancer Multi-Omics

**Figure 4:**
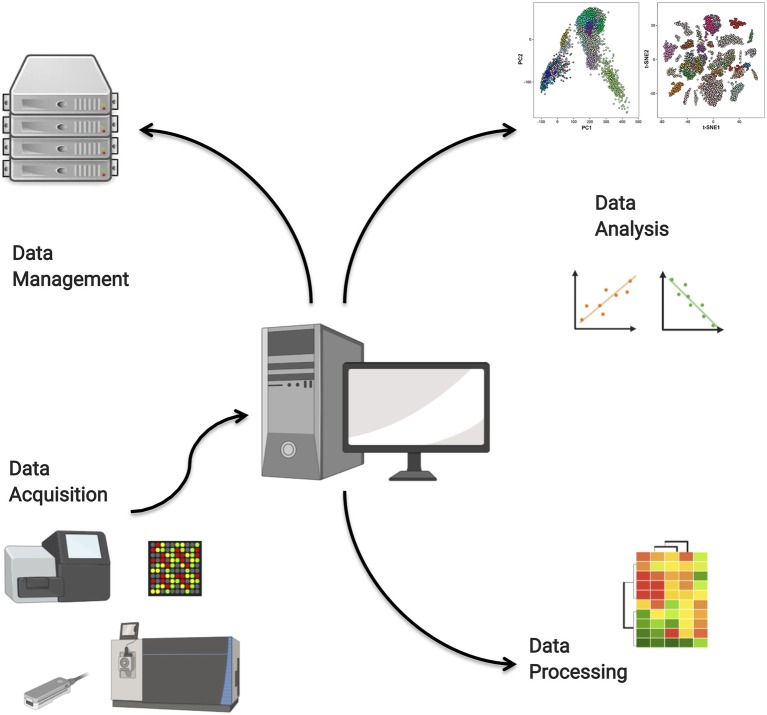
Computational tools are needed for high-throughput data acquisition, data management in repositories, data processing, and high-end analysis.

We have identified four main roles that computation plays in the analysis of high-throughput data. These are the raw data acquisition from high-throughput instruments; the processing of raw data to quantitative data; the storage and management of massive omics data, for instance in remote repositories; and finally the deployment of data analysis models. These roles are illustrated in Figure 4. In this section, we will discuss select aspects of each of these roles.

### 3.1. Data Acquisition and Processing

**Figure 5:**
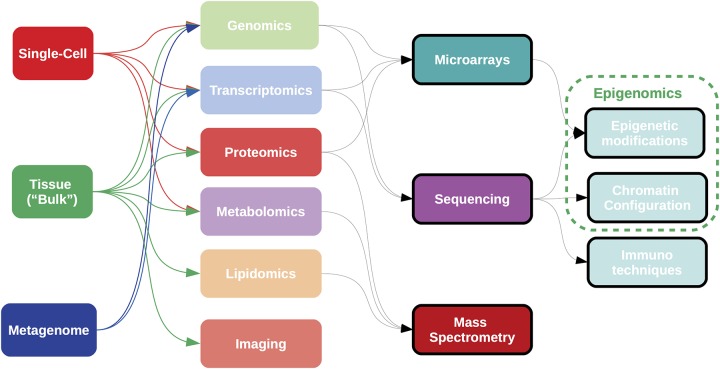
Samples for omics analyses can be obtained from “bulk” tissue, single cell data, or heterogeneous populations, such as metagenomes. Most current omics data are generated using technologies either array-based, sequence-based, or mass spectrometry-based; although high-throughput imaging data is becoming important in the clinical setting. Complementary techniques exist for the analysis of epigenetic states. Each combination of sample type, omic measurement and analytical technology requires a specific bioinformatic pipeline for data acquisition and processing.

The acquisition, processing, and manipulation of omic data generated in high throughput experiments requires, due to the very nature of these experiments (see Figure 5), the use of specialized bioinformatics pipelines. As the complexity of these datasets increases due to the natural evolution of these technologies, so do the associated challenges evolve (20). Bioinformatics workflow management systems can be used to develop, maintain, and foster reproducibility of a give pipeline or workflow. Examples of these systems include Galaxy (21), Snakemake (22), Nextflow (23), and the general purpose Common Workflow Language (24).

It should be noted that a large number of tools for omic data analysis are available as packages for the R language contained in the *Bioconductor* project (25), a repository of bioinformatics open source software. It is important, however, to acknowledge the existence of other software ecosystems, such as the *Biopython* project (26). Although the number of packages in Bioconductor is greater than that found in Biopython [see for instance (27)], the main takeaway should be that there is a large number of tools available to researchers that can be used in any combination suitable for their research question.

#### 3.1.1. Genomics

The oldest of the omic technologies, genomic analyses focus on the genomic sequence and its variations: insertions, deletions (INDELs), single nucleotide variations (SNVs), copy number variations (CNVs), and so forth. The relationship between genomic alterations and cancer is well-known (28).

Microarrays have long been used for genotyping. Although specifics of microarray technology may vary across manufacturers, most modern DNA microarrays can be analyzed using well-established tools available in the *Arrays* (29). Such tools can handle arrays for different genotyping tasks, including SNP and copy number assays [for instance, copy number detection from exome sequencing using *CODEX* (30)].

Although DNA microarrays remain in use, next generation sequencing (NGS) technologies are quickly becoming commonplace. The analysis of NGS data entails a workflow that involves sequence acquisition and alignment to a reference genome, A number of downstream analysis pipelines can follow; for instance, a variant discovery workflow would involve variant calling, filtering, annotation, and prioritization (31). The first step to analyze NGS data is to use a sequence aligner tool on the sequence data (stored in FASTQ format). Some popular aligners are the stand-alone *BWA* (32), *Bowtie* (33), *Bowtie2* (34), and *SNAP* (35), with aligned sequences being stored in SAM (Sequence Alignment Map, text-based) or BAM (Binary Alignment Map) files. These aligned sequences are the input for downstream genotyping analyses (36, 37).

Such *standards* are indeed a matter of state-of-the-trade in the academic research community indeed. Regarding pipelines approved by regulatory instances, there is in fact an official FDA guideline document to this end: “Considerations for Design, Development, and Analytical Validation of Next Generation Sequencing (NGS)—Based *in vitro* Diagnostics (IVDs) Intended to Aid in the Diagnosis of Suspected Germline Diseases” available for download at https://www.fda.gov/media/99208/download. The Guideline document (99208) actually refers to a Software Documentation Guideline: “General Principles of Software Validation; Final Guidance for Industry and FDA Staff” which is however quite outdated (last revised January, 11, 2002) (https://www.fda.gov/media/73141/download). Some NGS tools however are actually available as a web service at https://precision.fda.gov/. For a review on these guidelines and tools see (38).

#### 3.1.2. Epigenomics

With the recent advent of high-throughput omic technologies to probe chemical modifications in the tumor genomes it has become more and more evident that such epigenomic modifications are present and likely play relevant roles in many cancers. These variations include DNA methylation and histone modifications, both in oncogenes and in other cancer-associated genes. Mutations in genes involved in epigenetic regulation have also been found in several tumor types. The computational analysis of epigenomic data may provide us new insights about cancer initiation and progression. More relevant perhaps, such studies will pave the way for a more efficient identification of genetic and epigenetic biomarkers for diagnosis, prognosis or response to therapy. These in turn, may accelerate the development of novel therapeutic approaches.

Epigenomics often presents another view of functional processes complementary to that of genomics. Sometimes epigenomic techniques even allow for a better understanding of genome-associated phenomena. Such is the case of high-throughput immunoprecipitation assays, such as ChIP-Seq. ChIP-Seq and other experiments based on the analysis of short reads show the effects of multi-reads, i.e., reads that map to more than one genomic region. Determination of the origin of such multi-reads indeed results critical for the accurate mapping of reads to repetitive regions, such as copy number variants (39, 40). Current computational approaches have been refined to cover up for this phenomenon even at the single-cell level (41).

The epigenome contains the set of potentially inheritable chemical modifications of DNA and histone proteins that can control gene expression activity (42). There are several mechanisms which are contained within the epigenomics concept, each requiring a different high throughput molecular technique for its measurement. Each of these techniques, in turn, requires the use of a dedicated set of computational tools. These include:

- DNA methylation: The methylation state of a DNA region can alter its transcriptional activity. This state can be measured using either array-based methods or sequencing methods, such as the popular whole-genome bisulfite sequencing (WGBS) (43). Data from array based methods can be processed using the aforementioned array packages, along with dedicated packages, such as *methylationArrayAnalysis* (44). Similarly, those obtained using sequence-based methods can make use of dedicated tools, such as the *bsseq* (45) or *methyAnalysis* (46) packages.
- Chromatin remodeling: Regions where nucleosomes are sparse and physical access to the DNA sequence is enabled are identified as open chromatin. Chromatin accessibility is a dynamical and complex framework modulated by diverse elements, including nucleosome occupancy and turnover rate, histone modifications, ATP-dependent chromatin remodeling complexes and even TF binding (47, 48). Open chromatin has emerged as indicative of transcriptional regulatory potential or activity across the human genome because most of the TFs analyzed to date bind within open regions (49). Chromatin architecture is modified by changing its accessibility affecting gene expression rates. This remodeling can be controlled by histone modifications, which include acetylation, methylation, ubiquitination, and SUMOylation, among others. Overall chromatin accessibility can be also measured by techniques, such as ATAC-seq (50), a high throughput NGS technique to assess genome-wide chromatin accessibility. Due to the characteristic biochemical design of the assay ATAC-seq is a faster and more sensitive analysis of the chromatin accessibility than other alternatives, such as DNase-seq.
  ChIP-seq (51) data is used to identify genomic locations with an overabundance of proteins of interest; such identification uses the so-called *peak callers* (52, 53). These include *SICER2* (54), *PeakRanger* (55), *GEM* (56) *MUSIC* (57), *PePr* (58), *DFilter* (59), and *MACS* (60); benchmarks for these algorithms can be found at https://github.com/skchronicles/PeakCalling.
  *MACS* is a popular peak caller that uses dynamic Poisson distribution; its successor, *MACS2* (61), improves the algorithm to, amongst other things, make it more suitable for calling differential regions. Differential binding analysis (that is, identifying sites in which exhibit a different binding behavior between biological conditions) can be useful to identify relevant regions that may be driving cancer phenotypes, using ChIP-seq data. Tools for this task include *DiffBind* (62), a package that provides functions to handle the results of peak set callers, such as *MACS*. Another tool for this task is *csaw* (63), useful for de novo detection of differentially bound regions using a sliding window approach. In-depth comparison of differential ChIP-seq analysis tools can be found in (64).
- Chromosome conformation: The three-dimensional organization of the genome allows for interactions between regions that are distant in terms of sequence, even belonging to other chromosomes. These higher-order chromosome structures are a current area of research in oncology (65). Chromosome configuration capture techniques are able to quantify interactions between genomic loci. These *C-techs* are based on the original 3C, *Chromosome configuration capture* (66); able to quantify interactions between a single pair of loci. It was followed by: 4C (*Chromosome configuration capture-on-chip*) (67), which captures interactions between one locus and all others; 5C (chromosome conformation capture carbon copy) (68), which captures all interactions between two sets of loci; and Hi-C (high-resolution chromosome conformation capture) (69, 70) to detect interactions between all possible loci pairs. Development of computational analysis tools for chromosome conformation capture data is ongoing, although there are available packages for the detection of significant interactions for all these technologies (71–73).

It has been known for some time that higher order chromatin arrangements are associated with chromosomal alterations in cancer. For instance, it has been argued that spatial chromosome conformation and negative selection may be powerful driving forces behind somatic copy number alterations (74). More recently, chromatin conformation capture has allowed the identification of putative pharmacological targets in breast cancer (75). Genomic loci interactions may even affect the expression of biomarkers related to hallmarks of cancer, such as hypoxia (76).

Packages, such as *methylPipe* and *compEpiTools* provide an integral platform for the comprehensive and integrative analysis of the first two classes of epigenomic data (77), whereas *ATACseqQC* (78) is a package offering quality control tools for ATAC-seq data, while *esATAC* (79) offers a whole analysis pipeline and the *GenomicInteractions* package (80) offers a complete framework for the analysis of chromosome conformation data.

#### 3.1.3. Transcriptomics

Transcriptomic analyses are used to measure the presence and abundance of RNA in a given physiological context (81). Perhaps the most common application of transcriptomic technologies is to measure gene expression. The gene expression profile of a phenotype can be used as a barcode of its biological state. Such barcodes can be compared, through differential expression analyses, to pinpoint cellular changes in cancers (82). The expression profile is the product of the gene regulatory program encoded in the genome and the epigenome. By measuring gene expression, we are indirectly capturing the regulatory changes that are at the core of the disease.

The development of gene expression microarray technology (83) has made gene expression measurement more technically and economically viable than the measurement of protein abundance. Therefore, methods for the measurement of biological activity (i.e., pathways) have been developed with transcriptomic data in mind (84). Studying the molecular phenotype of cells via transcriptomics has become an invaluable tool providing a proxy to the functional state of cells and its regulatory interactions, both in cancer (85, 86), and in healthy phenotypes (87). Nevertheless, it should be noted that the correspondence between gene and protein abundance is far from perfect (88), which highlights the need for multi-omics.

Beyond gene expression, whole transcriptomic analyses involve the measurement of non-coding (nc) RNA, such as micro-RNA (miR), long non-coding RNAs (lnc-RNA), small nucleolar, Piwi-interacting, enhancer RNAs, among others (89, 90). The role of these transcripts, particularly in terms of their contribution to the regulatory program, remains an active area of study.

As previously mentioned, transcriptomic technologies are one of the most developed omics, second only to genomics itself. Measurement of transcript abundance can be done using either expression microarrays or RNA-sequencing (91, 92). Each methodology has technical considerations, but the general steps for their analyses are similar: acquire and preprocess data, removing technical artifacts; quality control; and data normalization. The resulting data can be represented as an expression matrix: an NxM matrix where rows represent transcripts, and columns represent samples (or observations). It should be noted that most expression pipelines are oriented toward differential expression analyses [see for instance (93)]; this should be taken into account in case that is not the intended use-case.

Starting points for RNA-seq data analysis include either alignment based methods, such as *Bowtie* (33), and *STAR* (94), or alignment-free methods, such as *kallisto* (95) and *Salmon* (96).

Cancer-related omic experiments often rely on specific, tailor-made analytics. One instance of this is provided by alignment-free RNA-Seq analysis methods, such as the ones performed by kallisto, Salmon, etc. Alignment-free methods (AFMs) are particularly well-suited to study cancer transcriptomics to look up at the role and abundance of fusion transcripts that may give rise to chimeric proteins (97, 98). Another reason behind the use of AFMs is that it is known that different RNASeq pipelines present differences that may be important when analyzing cancer genomes and transcriptomes (99, 100).

Further require different tools for quantification, quality control, and normalization of expression data. For instance, a popular pipeline is composed of the aforementioned *Bowtie* as a short read aligner, *TopHat* (101) for the identification of slice junctions, *Cufflinks* (102) for transcriptome assembly and differential expression analysis, and *CummeRbund* (103) for result exploration; it should be noted that, while this pipeline is still widely used and maintained (e.g., Bowtie2 latest release was 02/28/20), other approaches are been gradually embraced by the community (104); for instance, the *HiSat2* (105), *StringTie* (106), and *Ballgown* (107).

In the case of tools like STAR, we need to be aware that fusion detection using STAR-fusion is mainly limited by the length of single-end reads. The STAR-fusion wiki (https://github.com/STAR-Fusion/STAR-Fusion/wiki) indicates the need for at least 100 base length. In the case of other approaches, such as FusionHunter (108) the authors recommend to align to a pseudo-reference and discard junction spanning reads with <6 bp matches on either gene. *Arriba* is a relevant tool to call for gene fusions, based also in the STAR-alignment (https://github.com/suhrig/arriba/). Arriba was the winner of the DREAM SMC-RNA Challenge (https://www.synapse.org/#!Synapse:syn2813589/wiki/401435) (109).

An advantage of the modular design of these pipelines is that it is possible to combine tools from different workframes, depending on experimental and analytical needs: For instance, *Salmon* provides tools to connect with differential expression tools, such as *DESeq2* (110), *edgeR* (111), *limma* (112), or *sleuth* (113). A detailed discussion of these methods is beyond the scope of this article; please see Conesa et al. (114) for an in-depth review.

#### 3.1.4. Proteomics

Proteomic analyses are used to identify and quantify the set of proteins present within a biological system of interest (115). The study of cancer proteomes is promising as a way of identifying biomarkers and therapeutic targets (116). This is not surprising: proteins are the molecular unit from which cellular structure and function arises.

Historically, high throughput proteomics technologies have developed at a slower pace than genomics and transcriptomics technologies. Microarray approaches to proteomics have been developed, with varied levels of success and applications (117, 118). However, the bigger breakthroughs have come through the use of mass spectrometry (119).

Various steps of proteomics analysis involve data analysis (120). During data acquisition, the detected molecular fragments must be identified. This is often done by comparing fragments to databases in real-time (121, 122). Later, the assembly of proteins from identified peptide fragments requires another set of computational methods (123). The development of such methods remains an active area of research (124, 125). The *Bioconductor* offers a streamlined set of tools for the management of proteomics data, from data processing to functional analysis (126). Another alternative for protein quantification is the *maxquant* toolset (127).

#### 3.1.5. Metabolomics and Lipidomics

Metabolic alterations are important contributors to cancer development (128). Cancer metabolomics has become an important research topic in oncology (129), with the promise of providing novel insights on cancer development and potential therapeutic options. Lipidomics is actually a subset of metabolomics (130). The study of cancer lipidomics may lead to the identification of biomedical important findings, such as novel biomarkers (131).

Like proteomics before, metabolomics and lipidomics studies have been possible thanks to the use of mass spectrometry. The analytical considerations for the extraction and quantification of these types of compounds have some differences to those used for proteomics. This is expected, as the chemical nature of metabolites and lipids are fundamentally different (132, 133). In turn, bioinformatic and chemoinformatic approaches to high-throughput metabolite profiling exhibit some modifications (134).

Analysis frameworks for metabolomic and lipidomic data are currently available. The *metab* package (135) provides an analysis pipeline for metabolomics derived from gas chromatography—mass spectrometry data. The *metaRbolomics* package (136) is a general toolbox that goes from data processing to functional analysis. Finally, the *lipidr* package (137) is a similar framework focused on lipidomics data.

#### 3.1.6. Unraveling the Complexity Within Samples: Single Cell, Imaging, Microbiome

The aforementioned technologies were all developed for the detection and quantification of analytes extracted from a complex biological matrix, obtained from tissue, plasma, or a similar fluid. As such, the data from these omics is an aggregate of the different cellular contexts present in the sample. The environment within and surrounding cancer tumors is notably heterogeneous (138, 139). There is knowledge to be gained by recovering the omics diversity within samples.

Cancer is an extremely heterogeneous disease at the cellular and molecular level. Tumor heterogeneity caused by the concurrence of multiple cell lineages and differentiation stages, determined to an extent by the processes of clonal evolution. This has led to an early adoption of single cell analysis techniques. The case of single cell sequencing to study the genomic and epigenomic features of the different cell populations within a tumor by considering the characteristics of individual cells has revealed as an appealing approach to deal with said cell-to-cell variability (140–142).

Cancer cell heterogeneity also exists beyond the genome. Tumor evolution under complex environmental scenarios often leads to variability in epigenetic modifications. Single cell sequencing and imaging techniques have proven to be quite effective to characterize cellular plasticity induced by epigenomic phenomena (143). Aside from scMethSeq, and scDNAse Seq, other techniques, such as single-cell chromatin accessibility assays are starting to shed light to how epigenomic subpopulations in cancer may have the potential to impact tumor features, such as drug sensitivity and clonal dynamics (144).

*Single-cell* omics analyses rely on experimental techniques for the isolation of single cells from a sample, using microfluidics or fluorescence-activated cell sorting methods (145). Single-cell RNA-seq (scRNA-seq) is currently the most developed high-throughput omics technology for individual cell analysis (146).

Data from scRNA-seq experiments can be thought to be very similar to so-called “bulk” data. Data from scRNA-seq is, in fact, sparser, more variable, and with more complex expression values distributions. As such, data analyses techniques may need to account for different assumptions than their “bulk” counterparts (147). Again, the development of these novel bioinformatics tools is an active area of research (148). The *Bioconductor* ecosystem has a complete framework for the analysis of scRNA-seq from low-level (149) to functional analyses (150). *Scanpy* (151) provides a toolkit for single-cell gene expression analysis in a Python environment. Another single-cell genomics toolkit is *Seurat* (152) for R.

Integration of single-cell RNA-seq with other profiling tools is an important research area (153); as along with *single-cell*, there are other technologies that can provide a more complete picture of the cancer heterogeneity. High throughput imaging techniques (154) can be generated and computationally analyzed (155, 156). Imaging techniques can be used along with omics to recover the spatial distribution of molecules within cells and throughout tissues. Tools, such as *CellProfiler* (157) allow for a high-throughput analysis of data. Imaging techniques can be combined with single-cell methods: for instance, *MERFISH* can simultaneously measure copy number and distribution of RNA in single cells (158); *Slide-seq* (159) can measure transcriptomes at a high spatial resolution.

Space-resolved transcriptomics or spatial transcriptomics (ST) is a set of *in situ* transcript capturing methodologies aiming at quantification and visualization of gene expression patterns in individual tissue sections or regions. ST methods have indeed revealed relevant tissular phenomena linked to tumor evolution and in some cases have been able to allow the prediction of clinical outcomes in, for instance, breast cancer subtypes (160).

ST mapping of prostate tumors, on the other hand, have resulted key in the identification of gene expression gradients in stroma adjacent to tumor regions. This in turn has resulted in patient re-stratification based of tumor microenvironment features (161). A similar approach has been taken to trace tumor advance in malignant melanoma (162). A combination of ST with scRNASeq has led some researchers to propose the concept of a “tumor atlas,” a roadmap to navigate tumor spatial and cellular heterogeneity (163).

Multi-omic analysis is not devoid of technical and logistic conundrums. Perhaps the most obvious is the availability of the different sample types from a single source in the same experiments. Cell cultures may provide a way out to this problem, however *in vitro* conditions are often not resembling some aspects of interest in complex phenotypes, such as cancer. In recent times, three dimensional cell culture techniques have allowed the design and development of more *realistic* models, such as the case of organoids and tumoroids. These models may represent a good compromise between cell line studies and biopsy-captured tissue experiments (164). Multi-omic approaches are starting to be applied on lab-grown organoids with relative success (165, 166). In order to analyze such data some novel computational tools are being developed and adapted (167).

The role of the immune system in cancer response is another area of active research. CITE-Seq is an RNASeq method that incorporates epitope analysis thus leading to semiquantitative information regarding surface protein abundance via antibody assays, even at the single cell level (168). This novel technique is starting to be applied to provide the answer to fundamental questions in oncology, such is the case of tumorigenesis (169)

Finally, the role of the microbiome in cancer is being recognized (170); the integration of metagenomic, and perhaps *meta-omics* data (171), could provide key insights into cancer pathogenesis and therapeutics.

### 3.2. Data Management

The push for open data in the field of biomedical genomics since the gestation of the Human Genome Project has led to the emergence of a rich Genomic Commons (172). Making data available in public repositories makes for faster scientific discovery, although there are challenges to be overcome, both ethical/legal (173), and technological.

**Figure 6:**
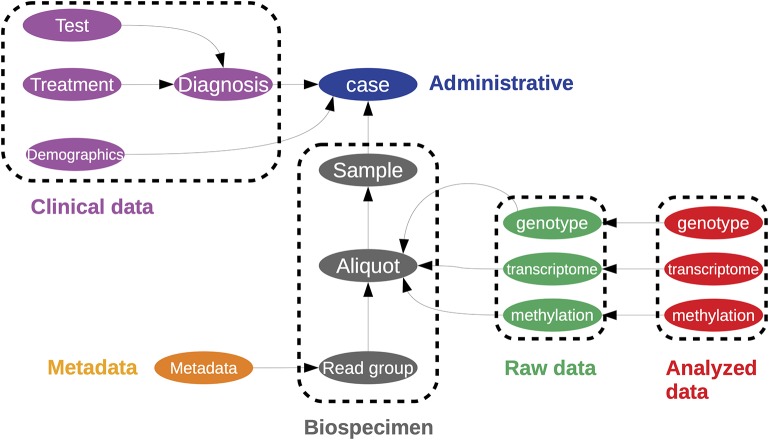
A representation of the data structure used in to store the Cancer Genome Atlas within the Genome Data Commons. This is represented as a directed graph. This is a simplified illustration of the one found at https://gdc.cancer.gov/developers/gdc-data-model/gdc-data-model-components.

Challenges of data management include defining the type of data to be stored and how to store it; the policies for data access, sharing, and re-use; and long term archiving policies (174). Arguably, the most successful repository of cancer multiomics is NIHs Genome Data Commons (GDC) (175). The Genome Data Commons contains all data generated by the Cancer Genome Atlas (TCGA) project (176); although it should be noted that not all data is publicly accessible. The data is organized as a directed graph comprised of interconnected entities (Figure 6), with each entity having an associated set of properties and links. Data is publicly accessible either through the *gdc-client* command line tool, the REST API for programmatic access to the database, or through dedicated packages, such as *rtcga* (177). A recent account by *The ICGC/TCGA Pan-Cancer Analysis of Whole Genomes Consortium (PCAWG)* of these resources and analyses is presented in (178). Furthermore, a larger collection of datasets can be accessed through the Broad Institute's *Firehose* (http://gdac.broadinstitute.org/); cloud computing enabled data access is provided through the Cancer Genome Collaboratory (https://cancercollaboratory.org/).

The impact of TCGA at the forefront of multiomics research is inarguable. As a publicly available resource, it provides data for method development and validation. This is used by a lot of current projects. However, there are other datasets with either single layer or multiomic datasets that can also be integrated. And wetlab researchers still carry out their projects, contributing to the cancer multiomics community. Integrating data from both, local experimental projects and large collaborative endeavors, such as TCGA is indeed a common practice in many places, such as our institution, the National Institute of Genomic Medicine in Mexico. Doing so allows to contrast specific hypothesis for the different research groups with the statistical power obtained via the much larger datasets generated by international multicentric collaborative projects.

As mentioned, it is possible to extract a lot of knowledge from the systematic re-analysis of data available in large public datasets. Perhaps, the more comprehensive of these databases is the one by the TCGA/Genome Data Commons/International Cancer Genome Consortium, TCGA. Retrieving the data via their Application Programming Interface (API) (https://gdc.cancer.gov/developers/gdc-application-programming-interface-api) demands some familiarity with command line tools and coding that may be beyond of most non-bioinformaticians. The project's data portal (https://portal.gdc.cancer.gov/) provides easy to use interfaces, but may be limited on its application to broader analyses. To date there is a number of commercially available platforms that provide a gentler access to the TCGA data. Such is the case of Qiagen's OncoLand database (https://digitalinsights.qiagen.com/products-overview/discovery-insights-portfolio/content-exploration-and-databases/qiagen-oncoland/) and the cloud-based analytics solution Seven Bridges (https://docs.sevenbridges.com/docs/tcga-data). A limitation, aside from being subscription based alternatives that require a payment is that they are not customizable, which means that not all possible (nor desired) analysis may be performed.

There are, however a number of resources not only to access the data but to actually perform different levels of downstream analysis. Such is the case of imputation approaches to missing data in the TCGA database (179) (https://github.com/mrendleman/MachineLearningTCGAHNSC-BINF/).

Perhaps, the best combination of usability and versatility is present in the TCGA Workflow suite available as an R/Bioconductor package (180) (https://www.bioconductor.org/packages/release/workflows/vignettes/TCGAWorkflow/inst/doc/TCGAWorkflow.html).

## 4. Computational Tools for Multi-Omics Data Integration

**Figure 7:**
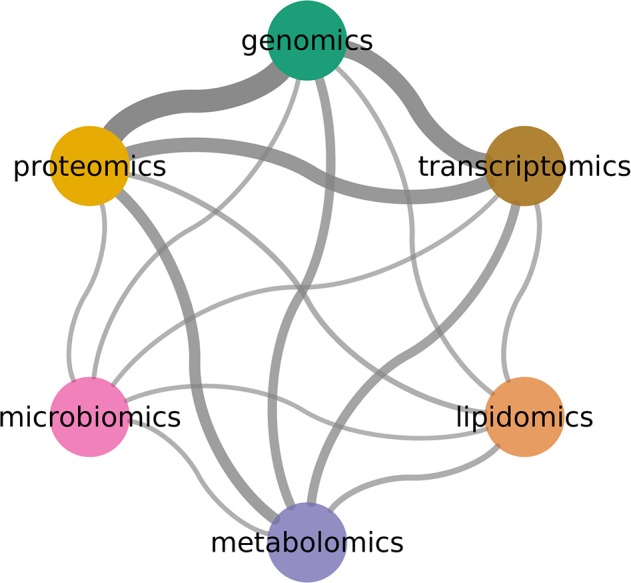
Combinations between omics technologies. Width indicates number of co-occurrence in literature. Genomics, transcriptomics, and proteomics are the most common pairs.

An often-asked question is why try to integrate multiple omics technologies using complex models. Perhaps the simplest argument is that the biological phenomena is not comprised of independent layers of biological features: integrative models will be, due to this simple fact, closer to the system of study. As omics technologies become available, researchers have used them together to try and capture a better description of the phenomena (see Figure 7).

Improving our current cancer diagnostic capabilities is a major goal of biomedical research: the role of molecular technologies in the development of these tools has long been recognized (181). It is expected that multi-omic integration is able to provide better predictive tools than single molecular technologies, due to the fact that each technology is capturing just a slice of the whole complex pathological system; multi-omics data are expected to be of value for both basic and clinical research, as long as they are able to recover biological insights beyond those obtainable from the simple addition of each analysis layer (182, 183).

It may soon become evident that the formalisms that can lead to such level of description are, by necessity, complex (184). A remaining question is what multiomic combinations are able to achieve better diagnostic results. Selecting this optimal omics combination is not trivial, since there are practical constraints (such as economic and technical limitations) in the clinical setting in which such diagnostic tools are to be deployed (185). Computational tools and bioinformatic approaches play an important role in the design of such studies. A list of such tools is presented in Supplementary Materials as Table 1.

### 4.1. Multi-Omics Data Representation and Preparation

The success of a computational method could arguably be influenced by the design principles implemented in its data representation. The *MultiAssayExperiment* package (186) provides an eponymous data class to contain multi-omics experiments. Like other *Bioconductor* classes, *MultiAssayExperiment* is object-oriented. It can contain the information of different (multi-omics) experiments, linking features, patients, and experiments. Furthermore, by sharing design principles with the rest of the S4-*Bioconductor* classes, it is highly interoperable.

An important issue with large scale multi-omics studies is the problem of missing and mislabeled samples. Whether by technical limitations or human error, the samples associated with a given patient may not have all measurements; or samples from two different patients may get mixed-up. There are packages available to handle these problems. The *missRow* package (187) can be used to handle missing data, combining multiple imputation with multiple factor analysis. The *omicsPrint* package (188), in turn, can be used to evaluate data linkage through the use of linear discriminant analysis.

The *STATegRa* (189) project provides a framework for multi-omics data analysis and integration: these are *MixOmics* (190), descended from the *integrOmics* project (191); and just like the *Bioconductor* project, the major advantage of such projects is the increased interoperability due to the sharing of design principles. For instance, within the *STATegRa* project, there is an Experiment Manager System (192); *MOSim* (193) a tool that provides methods for the generation of synthetic multi-omics datasets. These datasets can be used for the benchmarking and validating of other integration tools; and an experimental multi-omics dataset (194).

### 4.2. Multi-Omics Data Integration as a Data Science Problem

**Figure 8:**
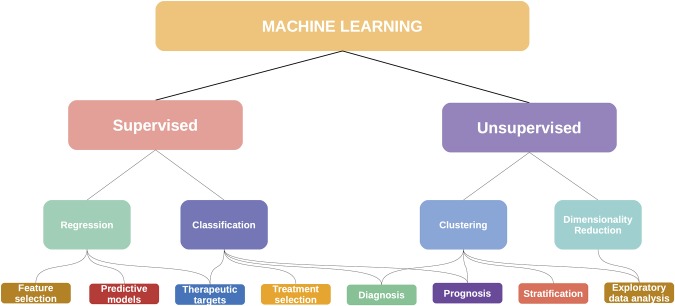
Machine learning has many applications in cancer and multiomics.

For this review, we approached these methods from a *data science* perspective, considering that each method is in essence solving a machine learning task (or set of tasks). In Figure 8 we show some of these mappings, although it should be noted that these categories may be fluid: an unsupervised clustering analysis can become the basis for a supervised classifier, with diagnostic and prognostic applications. This is the story of the PAM50 algorithm for breast cancer (195).

### 4.3. Exploratory Data Analysis

Exploratory data analysis (EDA) is a vital first step in omics analyses (196). Through EDA the nature of the data can be understood, allowing for better decisions at a further modeling step.

Unsupervised learning approaches can provide a hypothesis-free understanding of the data behavior. This will reflect the nature of the underlying biological phenomenon. *Unsupervised clustering analyses* attempt to group samples based on the similarity of their measured features. The assumption is that this unsupervised classification will recover relevant biological differences. Multi-omics can increase the efficiency of such approaches (197).

Multi Omic data analysis is often performed with the aim of unveiling non-trivial molecular and systemic interactions that are difficult or impossible to see if one relies on a single omic approach. However, since we are tacitly assuming that the different omic levels of description may have synergistic effects that are key to develop more accurate models of tumor biology. Since multi omic approaches may generate a plethora of interdependent data it is useful to design analytical strategies for dimensionality reduction, feature selection and integration of all this information.

Aside from intelligibility, there are additional reasons to make dimensionality reduction schemes, one of these is that a multi omic study combines different information sources, hence dramatically increasing the number of features, often keeping the number of samples constant, in order to preserve statistical power we need to rely only on the most informative variables (198–200).

Computational tools to this end have been developed, such as the following: https://www.bioconductor.org/packages/release/bioc/html/mixOmics.html https://bioconductor.org/packages/release/bioc/html/STATegRa.html For an extensive list of computational tools in the context of cancer biology, see (186).

One can make use of *dimensionality reduction techniques* in order to embed multi-omic data observations into a lower-dimensional space that can be used for either manual (i.e., visual) inspections or as the input for unsupervised clustering (or other analysis tools). Popular dimensionality reduction methods:

- Principal Component Analysis (PCA) is a classical (201) method based on an orthogonal transformation of the set of observations.
- T-distributed stochastic neighbor embedding (t-SNE) (202) is a method based on the minimization of the Kullback-Leibler divergence between the probability distribution of pairs of high-dimensional objects.
- The Uniform Manifold Approximation and Projection (UMAP) (203) is a non-linear technique in which data are projected into a Riemannian manifold.

*Data visualization* is an important part of EDA: the graphical representation of data can be sufficient for the identification of complex patterns (204). Visualizing high-dimensional biological data can be helpful from a purely data-driven point of view: for instance, to understand the variability within a phenomenon. Combinations of dimensionality reduction, data clustering, and visual inspection can be effective to identify subpopulations within a dataset. The most common visualization for these tasks is perhaps the scatterplot, but it is far from the only: for instance, *hexbins* (205) can be used to explore sc-RNAseq data, which can be useful to overcome overplotting problems related to the order in which points are drawn in the canvas.

Visualization can also be coupled with other biological information, for instance locating the genomic regions in which epigenomic features are found. Visualizations, such as the *Circos* plot (206) can be used for the detailed representation of multi-omics data and their location in specific genomic regions; The *omicCircos* (207) implementation is compatible with the standard data classes used in*Bioconductor*. The multiOmicsViz *multiOmicsViz* package is useful to visualize the effects of one omics layer to another, visualized in within the spatial chromosome context. The *Gviz* package (208) provides a full R graphics system solution for genome browser-style visualizations. Such representation is useful to represent the behavior of different experimental layers (as tracks) in a sequence context. For ChIP-seq data visualization, tools like *PAVIS* (209) may be used. Single Cell RNA-seq data visualization suites, such as *SingleCell Signature Explorer* (210) can be useful for exploratory analysis of such datasets. In the case of chromatin capture data, visualization toolboxes, such as HiBrowse (211), the Epigenome Browser (212), and Juicebox (213). For a thorough review of Hi-C visualization consult (214).

Common exploratory data analysis tools are implemented either in base R or as packages from CRAN (since their use is not necessarily limited to biological data). However, there are packages providing integrated EDA tools for multi-omics and oncology. The *OMICsPCA* package (215) provides omics-oriented tools for PCA analysis. The *CancerSubtypes* package (216) contains several data preprocessing, quality control, and clustering methods, focused on the identification of cancer subpopulations from multi-omics data. *Biocancer* (217) provides an interactive multi-omics data exploratory toolkit. The *omicade4* package (218) provides an implementation of multiple co-inertia analysis (MCIA), another dimensionality reduction technique; these tools were used for the integration of transcriptome and proteome data from the NCI-60 cancer cell line panel. The Multi-omics Autoencoder Integration (maui) is a tool for multi-omics data analysis for Python. It allows for latent factor model coupled with artificial neural networks for multiomics data integration. *iClusterPlus* is a Bioconductor package based on the original iCluster (219) algorithm for integrative cluster analysis combining different types of genomic data.

### 4.4. Statistical Models: Classificators, Predictors, and Feature Selection

Exploratory methods provide a useful description of biological phenomena. Nevertheless, in the oncology context, the identification of actionable elements is most desired, to generate translational value. The generation of models and feature selection strategies can lead to such results.

In this context, *statistical models* are computational (and thus mathematical) representations of the relationships between observed variables. These models can be useful to solve a given task based on some input data (220). Examples of these tasks include the *classification* of samples and the *prediction* of the state of a feature of interest.

Classification models have important biomedical applications (185). If a classification is able to discriminate between physiological states it can have translational use: A model that discriminates between health and disease has *diagnostic* utility; A model that discriminates between different disease outcomes has *prognostic* utility, which can be used for *stratification* purposes. Molecular classifiers have been quite successful in oncology: perhaps the best example being breast cancer (221). Classification models can be developed using *supervised* methods (that is, the model is trained with class information); but *unsupervised* methods, such as the previously discussed clustering, may be able to recover groupings that capture biological and clinical differences.

Predictive models can provide insights into the molecular mechanisms driving physiological states. These can reveal the interactions between different omics, as well as between individual biomolecules. Furthermore, predictive models can have translational applications, including their use in prognostic tools (222).

*Feature selection* consists in the selection of a subset of measured variables that are most informative: that is, they contribute the most for the model to accomplish its task. Proper feature selection is important for biomedical models (223), as (1) removing uninformative (“irrelevant” or “redundant”) features simplifies the model and increases its performance; and (2) a smaller set of features is less expensive to measure, increasing the translational potential of a given model.

Common applications of statistical models in the clinical context of cancer are the prediction of susceptibility, recurrence, and survival (223). Additionally, classification and association models are regularly used for the interpretation of molecular studies of cancer. For instance, biomarker discovery (224) is an often sought target for modeling based on biochemical and multi-omics analyses. This is an important area of study, since actionable biomarkers are not particularly common (225).

#### 4.4.1. Implementations and Use-Cases

Novel tools for the implementation of oncology models using model data are being released constantly. Many of these packages combine exploratory, supervised, and unsupervised tools, providing a wide range of analysis tools. *mixOmics* (190) is a self-described omics data integration project; it includes an eponymous package that provides different exploratory and integrative multivariate methods, including (independent) PCA, Canonical Correlation Analysis, Partial Least Squares regression (PLS), and PLS-Discriminant Analysis (DA). Part of the larger project is the *Data Integration Analysis for Biomarker discovery using Latent Variable approaches for ‘Omics studies* (DIABLO) framework, which has been used for the identification of a multi-omics signature of breast cancer molecular subtypes (226).

Other tools also follow this combined design principle. The *ropls* package (227), for instance, incorporates the tools for PCA, as well as (Orthogonal) PLS. Multi-Omics Factor Analysis (MOFA) is implemented in the eponymous package (228). This factor analysis model has been used for the unsupervised detection of groups in a leukemia dataset, and the selection of informative multi-omic features associated with oxidative stress. *OmicsMarkeR* (229) also provides a variety of classification and feature selection tools; originally developed for metabolomics, this tool has been used for the study skin cancer progression (230). Some packages include different classifier methods to generate an ensemble model; such is the case of *Biosigner* (231) which combines PLS-DA, Random Forests, and Support Vector Machines to select discriminant features across omics.

We agree with the assumption that multi-omics specific tools can improve workflows by adhering to a single design philosophy. However, we also agree that this is convenient, but not necessary. For instance, a diagnostic panel for pancreatic cancer was recently identified with a Random Forest implementation (232) using genomics, transcriptomics, and immunohistochemistry data. In another study, biomarker candidates for pancreatic cancer are identified using a Support Vector Machine on miRNA and gene transcriptomics (233).

Predictive models can be used to identify the contribution of one omics layer to the activity of another. For instance, *epigenomix* (234) uses Bayesian mixture models to integrate ChIP-seq and gene transcription data. The *Integrative analysis of Multi-omics data for Alternative Splicing* (235) package integrates expression, sQTLs, and methylation to provide mechanistic insights behind the manifestation of alternative splicing.

Predictive methods have been used to integrate multi-omics with other sources of big data, with publicly available implementations. The packages *rexposome* and *omicRexposome* (236) have been used to study the *exposome*, defined as the set of environmental exposures. Using multi-canonical correlation analyses and multiple co-inertia analysis, exposome-wide associations have been made to multi-omic data. The *OmicsLonDA* package (237) offers a method that uses linear mixed-effect models and smoothing spline regression models to identify time periods with differential omics levels. A highlight of this package is the consideration for the use of physiological measurements from wearable sensors, which may provide applications for *nowcasting*, the prediction of near-future states.

#### 4.4.2. Functional Aggregation

One could argue that analysis methods can be more informative if there is a way of associating the findings to the wider body of biomedical knowledge. Mapping omics data to functional features, such as pathways and functional genesets, is a strategy that can provide such readily interpretable results. *Functional enrichment* approaches, such as *over-representation analysis* (ORA) and *gene-set enrichment analysis* (GSEA), are effectively *feature extraction* methods that can be used as biologically relevant dimensionality reduction methods. The results of such methods can serve as starting points for more complex models, such as interactions among functions (238). For a detailed discussion of functional analysis, see (84).

The development of methods for effective functional enrichment based on multi-omics data is ongoing. *Multi-omics gene-set analysis* (MOGSA) (239) approaches the problem by using multivariate analysis, and using projections of data and genesets to lower dimensional spaces, to generate an enrichment score. *Massive integrative gene set analysis* (MIGSA) (240) takes a different approach, making independent functional associations for each omics layer (using ORA and Functional Class Scoring). Instead of providing an aggregated measurement, the functional associations of each layer are stored in a special data structure, allowing flexible analyses. This method has been used to functionally characterize breast cancer molecular subtypes from a multi-omics perspective.

Functional aggregation can be used as the basis for other data analysis tasks. In *pathwayPCA* (241), exploratory data analysis is done by analyzing the functional enrichment of each omics set separately, and aggregating them via consensus. This method was used to study heterogeneity in an ovarian cancer dataset. In the original work for the *Divergence analysis* (242) method for high-dimensional omics data analysis, the authors evaluate the effect of using functional aggregation for their data classification task. Functional aggregation methods are an important part of high-throughput drug initiatives, as can be seen by their prominence in the iLINCS platform (243).

### 4.5. The Network Paradigm

As we have stated throughout this work, biological phenomena are complex, interconnected systems. The data that we recover from high-throughput multi-omics is not isolated. Any biological system is not just the sum of its parts, but the sum of its biological elements *and their relationships*. With this in mind, the integration of high-throughput data within a network paradigm becomes appealing. Some advantages of a network approach to multi-omics integration are:

- A network representation of multi-omics data can be studied using all the foundations and tools of network science (244). Network topological parameters can be associated with important biological features; furthermore, dynamical processes can be modeled over networks.
- As previously noted, the functional level of biological description is fundamentally composed of molecular interactions. In other words, measurable functions can be thought to emerge from biological networks. Functional analyses can benefit from considering the way in which the participating molecules interact.
- The integration of interaction information can lead to more informative models (245).

A network perspective can enhance every aspect of the multi-omics analysis. For instance, mapping omics data to pathway networks can provide an opportunity to biologically contextualize the data. A classic tool for this is the *pathview* (246) package. The *Graphite* (247) package is a more flexible alternative, as it allows the visualization of pathways from different data sources, and provides proper graph objects that can be manipulated using network visualization tools. Recently, the *metaGraphite* package provided a major update to the original tool, effectively incorporating multi-omics through the addition of a metabolomics layer.

Network approaches can be used for classification and prognosis. For instance, the *micrographite* (248) package provides a method to integrate micro-RNA and mRNA data through their association to canonical pathways. This approach has been useful in identifying key micro-RNAs in myeloma (249), primary myelofibrosis (250), and ovarian cancer (251). *Mergeomics* (252) integrates data from genomic, epigenetic, and transcriptional association studies through a functional enrichment method, the results of which are used as the basis for a network construction; however, this tool has not been used in a cancer context. *pwOmics* (253) is another tool that leverages biological network knowledge to integrate multi-omics data. In particular, this tool is well-suited for the study of time series analyses.

While mapping data to predefined networks can be useful to gain a much-needed biological context, high-throughput technologies offer the opportunity to actually *infer* networks from the data itself. With such approach, data analysis problems can be transformed into network analysis problems. For instance, feature clustering becomes network module detection, which can be then used as the basis for a functional enrichment analysis (254).

While network reconstruction from omics data can be a powerful tool, it should be stated that every network reconstructed from data has an underlying hypothesis, which defines what the links between elements represent. This hypothesis should be at the center of any interpretation of the topological or functional associations recovered from a network. Furthermore, one must remember that comparison between reconstructed networks of different biological conditions will yield information about biological differences only if the method for network reconstruction does not deviate for each condition. For a discussion on this subject, see (255). This point is particularly relevant when discussing multi-omics data integration, as many of the network reconstruction methods available were developed for gene expression data. Proper validation of a method should be conducted before using it with other types of data.

There are some recent implementations of network reconstruction methods that have been developed with multi-omics data in mind. MAGIA^2^ (256) is a tool for the reconstruction of micro-RNA and transcription factor regulatory circuits; it has been used for the analysis of expression regulation in the NCI60 cell panel. The *Discordant* method (257) uses a mixture model to identify differential correlation: that is, statistical dependencies between feature pairs that are lost or gained from one biological state or another. This method has been evaluated for its use with different types of omics data. The *Netboost* (258) is a network reconstruction method infers statistical dependency based on multi-omics data, and uses a modularity approach to reduce dimensionality; the method has been used for the classification and survival analysis of acute myeloid leukemia data. *AMARETTO* (259) identifies pairwise relationships between different omic layers to select cancer driver genes. A module detection approach is used to construct a dimensionally reduced module network, which is further analyzed to identify molecular signatures.

Probabilistic network reconstruction is a powerful data analysis technique. In such a model, features are connected based on an information-theoretical similarity measure, such as mutual information, between their expression profiles. Unlike correlation metrics (260), mutual information can capture non-linear relationships between features, which makes it suitable for the analysis of transcriptomics (261). We have applied these methods for the reconstruction of micro-RNA and gene co-expression bipartite networks with minor adjustments; the analysis of such networks has yielded interesting insights on the nature of functional control by micro-RNAs (262). A current research interest the authors of this work is the extension of probabilistic network reconstruction for multi-omics reconstruction, in order to construct *probabilistic multilayer networks* (263) that can be studied using the recent tensorial formalism of multilayer networks (264).

### 4.6. Data Science in Biology—A Word of Warning

An important aspect of any data science project is the crucial role of both technical and domain specific expertise. The analysis of biological networks in particular can pose some complication for biological scientists not familiar with the field of network science; a network visualization may be presented as result, without an adequate evaluation of network topology or other structural and dynamic parameters. Similar behaviors can be found with other applications of data science tools.

A data-driven analysis without the participation of a domain expert risks the pursuit of non-relevant questions. On the other hand, even though a bioinformatics tool may be developed with an increased usability in mind, the level of complexity of both the computational method may require a deeper understanding of the algorithm's assumptions and limitations in order to reach valid results. With this in mind, it is evident that proper computational approaches to biological questions require a fundamental understanding of both in order to reach scientifically solid conclusions. In many cases, the key to achieve this is to strive for multidisciplinary approaches.

## 5. Conclusion

Cancer is the paradigmatic complex phenotype. We have been able to capture some of this complexity via experimental measurements with the different high throughput biomolecular technologies generically termed *omics*. Each single-technology derived data type has its own set of caveats and complexities. An additional challenge lies in the fact that each data type is able to account for a fraction of the large set of cancer aspects or features. Recent times have witnessed the development of new ways to gather and analyze these partial information layers together, under the name of multi-omics.

There are, however, multiple approaches to multi-omic computational modeling and integration, some of the most relevant have been described and discussed here. Our aim has been that of presenting the current state of the art of computational oncology tools for multiomic studies of complex cancer phenotypes. Novel developments in the multiomic computational analysis come from different fields, ranging from purely mathematical developments (263, 264), to machine learning and computational intelligence applications (179, 223), to single-cell sequencing and imaging studies (139, 145) and more. However, in our view, the development of methods to integrate all these different analytical approaches into intelligible and statistically robust frameworks will provide the field with unprecedented advances both in our understanding of cancer biology and in our impact in the clinical settings. The field is fast-growing and currently under development, with novel algorithmic approaches being constantly released, but we believe that the present account is a good starting point.

## Author Contributions

GA-J and EH-L contributed to reviewing and classifying the literature, structured the review, prepared the figures, wrote, and revised the manuscript. EH-L contributed to funding and general oversight of the project.

### Conflict of Interest

The authors declare that the research was conducted in the absence of any commercial or financial relationships that could be construed as a potential conflict of interest.
